# A prospective evaluation of inter-rater agreement of routine medical records audits at a large general hospital in São Paulo, Brazil

**DOI:** 10.1186/s12913-020-05495-w

**Published:** 2020-07-10

**Authors:** Ana Carolina Cintra Nunes Mafra, João Luiz Miraglia, Fernando Antonio Basile Colugnati, Gilberto Soares Lourenço Padilha, Renata Rafaella Santos Tadeucci, Ederson Almeida, Mario Maia Bracco

**Affiliations:** 1 Hospital Israelita Albert Einstein, Avenida Brigadeiro Faria Lima, 1188 - Jardim Paulistano, São Paulo, SP CEP 01451-001 Brazil; 2 Hospital Municipal Dr. Moysés Deutsch – M’Boi Mirim, São Paulo, SP Brazil; 3 School of Medicine, Universidade Federal de Juiz de Fora, Juiz de Fora, MG Brazil; 4 Centro de Estudos e Pesquisas Dr. João Amorim – CEJAM, São Paulo, SP Brazil

**Keywords:** Inter-rater agreement, Longitudinal agreement, Medical quality register, Audit, Gwet’s AC1

## Abstract

**Background:**

The quality of patient medical records is intrinsically related to patient safety, clinical decision-making, communication between health providers, and continuity of care. Additionally, its data are widely used in observational studies. However, the reliability of the information extracted from the records is a matter of concern in audit processes to ensure inter-rater agreement (IRA). Thus, the objective of this study is to evaluate the IRA among members of the Patient Health Record Review Board (PHRRB) in routine auditing of medical records, and the impact of periodic discussions of results with raters.

**Methods:**

A prospective longitudinal study was conducted between July of 2015 and April of 2016 at Hospital Municipal Dr. Moysés Deutsch, a large public hospital in São Paulo. The PHRRB was composed of 12 physicians, 9 nurses, and 3 physiotherapists who audited medical records monthly, with the number of raters changing throughout the study. PHRRB meetings were held to reach a consensus on rating criteria that the members use in the auditing process. A review chart was created for raters to verify the registry of the patient’s secondary diagnosis, chief complaint, history of presenting complaint, past medical history, medication history, physical exam, and diagnostic testing. The IRA was obtained every three months. The Gwet’s AC1 coefficient and Proportion of Agreement (PA) were calculated to evaluate the IRA for each item over time.

**Results:**

The study included 1884 items from 239 records with an overall full agreement among raters of 71.2%. A significant IRA increase of 16.5% (OR = 1.17; 95% CI = 1.03—1.32; *p* = 0.014) was found in the routine PHRRB auditing, with no significant differences between the PA and the Gwet’s AC1, which showed a similar evolution over time. The PA decreased by 27.1% when at least one of the raters was absent from the review meeting (OR = 0.73; 95% CI = 0.53—1.00; *p* = 0.048).

**Conclusions:**

Medical record quality has been associated with the quality of care and could be optimized and improved by targeted interventions. The PA and the Gwet’s AC1 are suitable agreement coefficients that are feasible to be incorporated in the routine PHRRB evaluation process.

## Background

Adequate medical record keeping is an essential part of good professional health practice that makes it possible to evaluate and improve the quality of health care. The use of medical records should extend beyond the medical management of patients: adequate records allow for improved coordination and continuity of care; serve as a learning tool; and help prevent and evaluate possible adverse events that may compromise patient safety in hospitals [1, 2].

In 2002 the Brazilian Medical Council established that patient record review commissions are mandatory for health services [3]. However, it does not establish criteria or guidelines for the evaluation or its reliability. When different raters assign the same value for each item being observed, it’s important to measure its inter-rater reliability (IRR), closely related to the inter-rater agreement (IRA) [4, 5]. Some review studies assessing adverse events have been shown to suffer from poor to moderate IRR [6, 7]. In addition, IRR is rarely described or discussed in research papers based on data extracted from medical records, and there are no standard methods for assessing IRR [8]. Moreover, time constraints and work overload are frequent situations faced by health staff performing tasks involving data management, resulting in low data quality that can affect managerial decision-making [2]. Therefore, the evaluation of suitable methods for data extraction from this source is essential [9].

When such studies employ multiple raters it is important to have a strategy to document adequate levels of agreement between them, and the Cohen’s Kappa coefficient (κ) is a well-known measure [10]. However, it is affected by the skewed distributions of categories (the prevalence paradox) and by the degree to which raters disagree (the bias problem) [11, 12].

To correct those limitations, Kilem Li Gwet proposed a new agreement coefficient which can be used with any number of raters and requires a simple categorical rating system [13, 14].

The objective of this study was to evaluate the IRA of routine medical record audits and the impact of periodic discussions among raters in a large general hospital. The study also aimed to compare the estimates of the percent agreement (PA) to the Gwet’s agreement coefficient (AC1), to identify possible factors associated with the PA, and if agreement among the auditors is associated with the adequacy of the evaluated items.

## Methods

### Population and setting

This was a prospective longitudinal study conducted between July of 2015 and April of 2016 at the Hospital Municipal Dr. Moysés Deutsch (HMMD). HMMD is a large public general hospital (300 beds) located in the southern zone of the city of São Paulo, Brazil— an impoverished region encompassing approximately 600,000 inhabitants. The present study was part of a larger intervention aimed at improving the quality of patient care through a tailored integration strategy among health facilities in its Regional Health Care Network [15].

### Audit of medical records and review meetings

The HMMD maintains a routine auditing process that includes 13% of all medical records of patients discharged in the previous month, carried out by the Patient Medical Record Review Board (PMRRB). The PMRRB was composed of 24 nominated health professionals: 12 physicians; 9 nurses; and 3 physiotherapists. Half of them were staff coordinators for at least two years and a maximum of eight years. The auditors have an average professional experience of 14 years and 66.7% of whom were women. The audit is a time-consuming procedure because it competes with the patient-care tasks that these professionals are responsible for. Consequently, it is common for the audits to have been carried out by each PMRRB member in isolation from other members without any criteria alignment for rating the items on the audit chart, which compromises the quality of the entire auditing process. However, the patient’s medical charts were selected in a non-random way, lacking adequate representation across the achieved results, and compromising the accuracy of and ability to generalize these data.

The planned intervention used the Lean Six Sigma methodology, which is widely utilized to aggregate values in several HMMD quality improvement processes already a part of the work culture among the professionals [15].

The proposed actions included at least one team-leader from each HMMD clinical department, preferably its coordinator, which increased the PMRRB components, reducing the total medical charts to be reviewed by each member. The audit chart was refined by all members through discussions about the relevance of the information that should be registered by their health teams, answering the question: “Which information cannot be missed in the patient’s medical record?” The chosen items were then discussed to define the criteria to determine a rating as adequate, inadequate, incomplete, or not applicable (Table 1). For each item, a consensus was reached about its content as follows: Secondary diagnosis was considered adequate if it was registered at any time during hospitalization. A complete medical history should be rated as adequate only if the chief complaint, history of presenting complaint, medical and past medical history were present at the patient’s admission. A physical exam was adequate if registered by a physician encompassing a general and specific examination. Diagnostic testing was considered adequate if the results were transcribed, not merely checked as done. In the discussion meetings, all members were trained, and medical charts were presented on a screen, allowing all members to rate each chosen item by raising color cards as green (adequate), red (inadequate), and yellow (not applicable). Disagreements were discussed to reach a consensus. The medical records were filled out in an unstructured text.

**Table 1:** Audited items

Audited items	Rating Options
Secondary diagnosis	Adequate or inadequate
Chief complaint	Not applicable, adequate or inadequate
History of presenting complaint	Not applicable, adequate or inadequate
Past medical history	Not applicable, adequate or inadequate
Medication history	Not applicable, adequate or inadequate
Complete medical history	Not applicable, adequate, inadequate or incomplete
Physical exam	Adequate or inadequate
Diagnostic testing	Not applicable, adequate or inadequate

Finally, the patient medical records were randomly selected, weighted by the discharge proportion of each department.

The number of raters varying throughout the study is shown in Table 2.

**Table 2:** Number of audited medical records and raters over time

Audit period	Number of medical records for IRA	Number of raters
1. 2015 July	54	18
2. 2015 October	45	19
3. 2016 January	84	21
4. 2016 April	56	16

Every three months during the study period, in addition to the routine audits, five to six medical records were randomly allocated to the same two or three independent raters of the same professional category to evaluate the IRA. The study also included review meetings conducted every three months to align assessment criteria based on the results of the IRA evaluation and the auditing processes.

### Statistical analysis

The Gwet’s AC1 and PA were calculated to evaluate the IRA for each item over time and were compared through line graphs including 95% confidence intervals (CIs). The Gwet’s AC1 95% CIs [14] were calculated, while the PA were modelled by generalized estimating equations (GEE), without an intercept [16, 17]. The agreement measures were interpreted following the categories proposed by Altman [18].

Logistic GEE was used to model the PA of all raters over time, using the values of 1 for full agreement and 0 for some disagreement. Two designs were considered: combining all items, to attain global associations; and considering each item individually, to obtain more details. The analyses employed an exchangeable working correlation matrix, and items in a single medical record were considered to be correlated. The model included as independent variables: professional category, review meeting attendance, and time (audits 1 to 4). A forward stepwise approach was used for variable selection employing a *p*-value lesser than or equal to 0.200 in the unadjusted model, and lesser than or equal to 0.050 in the multiple-variable model.

To measure the association between the agreement and the adequacy of the items the Spearman correlation coefficient was applied. The agreement was measured as PA. The adequacy was measured as the percentage of “adequate” evaluations. Both were considered by item and time.

The analyses were performed with the R software version 3.2.2 [19] with geepack [20].

## Results

**Fig. 1:**
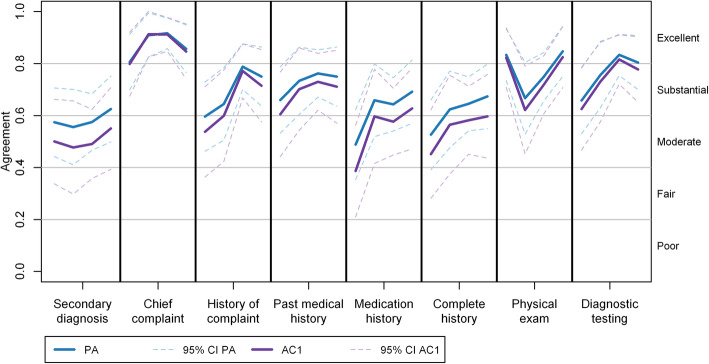
Estimated percent agreement (PA) and Gwet’s AC1 by audited item. CI: Confidence interval. AC: Agreement coefficient

The study included 1884 items from 239 records with an overall full agreement among raters of 71.2%. The estimated mean PA was found to be larger than the Gwet’s AC1 for all audited items (Fig. 1), however, these differences were not statistically significant and the evolutions of the two agreement coefficients were similar throughout the study period. Although a positive trend was found in the agreement of almost all items, their CIs did not indicate any statistically significant change over time. Additionally, the coefficients measurements grew closer as the agreement increased. During the study period, the greatest agreement was “chief complaint,” while the lowest one was “secondary diagnosis.”

The logistic GEE model that included all items (Table 3) found a statistically significant increase of 17% over time for the PA, but when at least one of the raters was absent from the review meeting, the PA decreased by 27%. Physiotherapists and physicians showed higher PA when compared to nurses.

**Table 3:** Estimated odds ratios (OR) for percent agreement. *N* = 1884 items from 239 records

	OR^a^ (95%CI)	*p* value	OR^b^ (95% CI)	p value
Time (audits 1 to 4)	1.20 (1.06–1.35)	0.004	1.17 (1.03–1.32)	0.014
Absent from meeting (Yes)	0.82 (0.60–1.11)	0.195	0.73 (0.53–1.00)	0.048
Professional category (physiotherapists)	1.49 (0.99–2.26)	0.058	1.66 (1.10–2.51)	0.016
Professional category (physicians)	1.45 (1.08–1.93)	0.013	1.44 (1.07–1.93)	0.015

^a^Estimates obtained by the unadjusted models

^b^Estimates obtained by the multiple-variable models

In the analysis by item, there was a non-significant positive trend for higher PA for “history of presenting complaint” while physicians presented a significantly higher PA over time when compared to nurses for “secondary diagnosis,” “medication history,” and “diagnostic testing.” Physiotherapists presented a significantly higher PA over time when compared to nurses for “medication history.” Finally, when at least one of the raters was absent from the review meeting the PA decreased by 60.5% for “diagnostic testing” (Table 4).

**Table 4:** Estimated odds ratios (OR) of percent agreement by item. *N* = 239 records

	OR (95% CI)	*p* value
Secondary diagnosis		
Time (audits 1 to 4)	1.02 (0.80–1.30)	0.878
Absent from meeting (Yes)	0.65 (0.35–1.23)	0.187
Prof. category (physiotherapists)	1.74 (0.72–4.22)	0.221
Prof. category (physicians)	1.82 (1.00–3.29)	0.048
Chief complaint		
Time (audits 1 to 4)	1.20 (0.80–1.80)	0.370
Absent from meeting (Yes)	0.71 (0.26–1.95)	0.507
Prof. category (physiotherapists)	1.22 (0.29–5.16)	0.786
Prof. category (physicians)	0.74 (0.30–1.83)	0.519
History of presenting complaint		
Time (audits 1 to 4)	1.31 (0.99–1.74)	0.056
Absent from meeting (Yes)	0.58 (0.28–1.19)	0.138
Prof. category (physiotherapists)	1.78 (0.68–4.61)	0.238
Prof. category (physicians)	1.56 (0.81–3.01)	0.182
Past medical history		
Time (audits 1 to 4)	1.14 (0.86–1.51)	0.375
Absent from meeting (Yes)	0.70 (0.34–1.44)	0.334
Prof. category (physiotherapists)	1.12 (0.43–2.91)	0.817
Prof. category (physicians)	1.35 (0.69–2.63)	0.378
Medication history		
Time (audits 1 to 4)	1.25 (0.96–1.62)	0.099
Absent from meeting (Yes)	0.83 (0.44–1.57)	0.569
Prof. category (physiotherapists)	4.25 (1.53–11.77)	0.005
Prof. category (physicians)	1.87 (1.02–3.41)	0.041
Complete medical history		
Time (audits 1 to 4)	1.22 (0.95–1.57)	0.118
Absent from meeting (Yes)	0.74 (0.38–1.42)	0.359
Prof. category (physiotherapists)	1.15 (0.47–2.82)	0.753
Prof. category (physicians)	0.96 (0.52–1.76)	0.893
Physical exam		
Time (audits 1 to 4)	1.07 (0.82–1.39)	0.616
Absent from meeting (Yes)	1.32 (0.68–2.58)	0.410
Prof. category (physiotherapists)	2.59 (0.70–9.57)	0.154
Prof. category (physicians)	1.02 (0.54–1.91)	0.950
Diagnostic testing		
Time (audits 1 to 4)	1.23 (0.91–1.66)	0.175
Absent from meeting (Yes)	0.39 (0.18–0.89)	0.024
Prof. category (physiotherapists)	1.52 (0.59–3.92)	0.387
Prof. category (physicians)	3.11 (1.53–6.30)	0.002

*Prof*. professional

The average adequacy of the items assessed in the first audit was 73.3%, increasing to 78.2% in the second audit, 76.3% in the third, and then falling to 72.1% in the fourth audit. Regardless of the time and type of item, when comparing the PA value with the percentage of adequacy, a Spearman correlation coefficient of 0.72 was found (*p*-value < 0.001).

## Discussion

A significant increase in the IRA among PHRRB members was found over time in routine medical record auditing processes when periodic evaluations of the agreement were performed and discussed by them. Supporting this finding, the absence of a member in a review meeting had a negative impact on the PA. In addition, the PA and the Gwet’s AC1 were comparable and presented a similar evolution over time. Complete medical history was a composite of chief complaint, history of complaint, past medical history, and medication history. It was considered adequate if all of them were complete. Thus, it showed a positive evolution in both PA and Gwet’s AC1 over time from moderate to substantial according to Altman’s categories [18]. Only the IRA of secondary diagnosis remained moderate. These findings may indicate the raters’ learning curve regarding the positive evolution of some variables across agreement ranges. Nevertheless, the degree of agreement is arbitrary, making it impossible to define an acceptable level [5]. Thus, the interpretation of these IRA values follows the main study objective, i.e., the raters’ concordance in a particular category.

The greater IRA among physicians and physiotherapists when compared to nurses may reflect some inconsistency across the evaluations that can be attributed to the raters’ selection, training, and accountability [5], and could be influenced by a misunderstanding about rating the “complete history” item.

The strategy applied to the IRA was feasible to be carried out in this real-world scenario, aggregating value to the auditing process and providing more accurate information that can be used by health leadership. The use of PA and Gwet’s AC1 for that purpose was successful because they demand a relatively small sample of PMRs to be audited by each rater and can provide two data consistency measures [5, 21]. Both of the used indices have reached acceptable levels of agreement [18, 22], according to study purposes.

Following and evaluating the progress of the agreement among raters of PMRs allows for setting up goals and identifying associated factors to improve the audit processes, but previously proposed models worked with continuous variables [23] or with the Kappa coefficient [24], so the use of PA and Gwet’s AC1 made it possible to model the agreement of more than two raters over time.

The increased IRA highlights the need for more careful planning and evaluation of medical record audits since this activity is closely related to health care quality and patient safety improvements efforts [8, 9].

Since the present study was conducted under real-world conditions and included different health providers as raters, this intervention has the potential to be applicable in other similar settings, taking into consideration that it was carried out in only one hospital that has a culture of evidence-based improvement interventions, during a short-term follow-up. Although this study did not include an evaluation of the impact in the quality of medical records, that should be the final goal of any routine audit. There was a strong association between agreement and adequacy of information registered in the patient health records, and although the study was conducted between 2015 and 2016, these results are still relevant given the lack of studies evaluating data quality of medical records auditing.

Furthermore, the literature on the quality of medical record keeping and IRA or IRR is scarce— reflected by the fact that no reviews on the subject could be identified— making the results of this study relevant to improve the body of knowledge in the era of data-driven institutions and big data from patient health records.

## Conclusions

Medical record quality has been associated with the quality of care and could be optimized and improved by targeted interventions. The PA and the Gwet’s AC1 are suitable agreement coefficients that are feasible to be incorporated in the routine PHRRB evaluation process.

## Data Availability

The dataset supporting the conclusions of this article is available to researchers who want to explore the data. To request, please send an email to ana.mafra@einstein.br.

## Abbreviations

AC1: Agreement coefficient
CI: Confidence interval
GEE: Generalized estimating eqs.
HMMD: Hospital Municipal Dr. Moysés Deutsch
IRA: Inter-rater agreement
IRR: Inter-rater reliability
OR: Odds ratio
PA: Proportion of agreement
PHRRB: Patient’s health record review board
